# Forehead monitoring of heart rate in neonatal intensive care

**DOI:** 10.3389/fphys.2023.1127419

**Published:** 2023-04-04

**Authors:** S. J. Stockwell, T. C. Kwok, S. P. Morgan, D. Sharkey, B. R. Hayes-Gill

**Affiliations:** ^1^ Optics and Photonics Research Group, Faculty of Engineering, University of Nottingham, Nottingham, United Kingdom; ^2^ Centre for Perinatal Research, Lifespan and Population Health, School of Medicine, University of Nottingham, Nottingham, United Kingdom

**Keywords:** photoplethysmogram, PPG, neonatal, heart rate, forehead, reflectance-mode, pulse oximeter Min.5-Max. 8, NICU (neonatal intensive care unit)

## Abstract

Heart rate is an extremely important physiological parameter to measure in critically unwell infants, as it is the main physiological marker that changes in response to a change in infant condition. Heart rate is routinely measured peripherally on a limb with a pulse oximeter. However, when infants are critically unwell, the blood supply to these peripheries is reduced in preference for central perfusion of vital organs such as the brain and heart. Measurement of heart rate with a reflection mode photoplethysmogram (PPG) sensor on the forehead could help minimise this problem and make it easier for other important medical equipment, such as cannulas, to be placed on the limbs. This study compares heart rates measured with a forehead-based PPG sensor against a wrist-based PPG sensor in 19 critically unwell infants in neonatal intensive care collecting 198 h of data. The two heart rates were compared using positive percentage agreement, Spearman’s correlation coefficient and Bland-Altman analysis. The forehead PPG sensor showed good agreement with the wrist-based PPG sensor with limits of agreement of 8.44 bpm, bias of −0.22 bpm; positive percentage agreement of 98.87%; and Spearman’s correlation coefficient of 0.9816. The analysis demonstrates that the forehead is a reliable alternative location for measuring vital signs using the PPG.

## 1 Introduction

Pulse oximeters are a crucial piece of equipment when monitoring critically unwell newborns in neonatal intensive care units (NICU). They are used to measure vital signs such as heart rate and blood oxygen saturation (SpO_2_). These vital signs are obtained by transmitting light through the body and investigating how the signal is modulated by the pulsatile blood flow. This modulation of light can be detected and is referred to as the photoplethysmogram (PPG). The PPG can be measured in two different modes, transmission and reflection (Jubran, 2015). Transmission mode PPG is used on areas where light can be transmitted through, such as the fingers, toes, and earlobes with the detector placed on the opposite side to the light source. Reflection mode PPG can operate on almost all parts of the body with sufficient cutaneous blood flow but with the detector placed alongside the light source.

The PPG signal originates from oscillatory changes in volume of the microvasculature as blood is pumped around the body by the heart. The PPG is regulated by several physiological factors, including but not limited to respiration (Nilsson, 2013), blood pressure (Elgendi et al., 2019) and neural activity (Khalid et al., 2022). These oscillatory changes can enable the extraction of multiple vital signs, such as heart rate, respiratory rate, and blood pressure. The PPG is used for a range of applications but here we focus on the newborn population. For details of other applications of PPG, we refer the reader to a recent review (Park et al., 2022).

Monitoring of the heart rate provides insight into the infant’s condition and how well they are responding to treatment. Although SpO_2_ provides oxygen status, heart rate is the first physiological marker to respond when an infant’s condition deteriorates or when treatment is successful (Wyckoff et al., 2020). Conventional pulse oximeters rely on the transmission of red and infrared (IR) light through the limb. Since the signal observed is due to pulsatile blood flow, the quality of the signal is reduced in the presence of poor peripheral perfusion. Reduced perfusion is common in the high-risk group of newborns due to a variety of reasons, including iatrogenic causes such as the use of inotropes (Dilli, Soylu, and Tekin, 2019) (Kluckow, 2018) or bloodstream infections (Verstraete et al., 2015). Hence, at a time when accurate heart rate measurement is crucial as the infant becomes critically ill, impaired peripheral perfusion can affect the ability of the conventional pulse oximeter to obtain reliable signals and subsequent heart rate measurements.

The space surrounding an unwell newborn in NICU can be especially limited with many wires and tubes being attached to the newborn and to the respective monitors to display the data. A sensor that uses wireless transmission to the bedside display not only reduces the number of wires around the newborn but also frees up vital space needed for important medical equipment, such as cannulas. Furthermore, the reduction in wires also helps parents establish skin-to-skin bonding (kangaroo care) with their newborn earlier (Bonner et al., 2017).

The new device used in this study is a reflection-based green light PPG probe mounted inside a cap placed on the forehead. This probe benefits from the strong absorption of green light (λ = 525 nm) in both oxygenated and deoxygenated haemoglobin, giving a large pulsatile signal. Furthermore, green light is applicable for heart rate detection since the deeper penetration depths provided by longer wavelengths, such as IR, is not necessary to measure the pulsatile blood flow in the microvasculature required to measure heart rate (Mejía-Mejía et al., 2022). As such, this optimises the measurement of the pulsatile signal of blood flow on the forehead.

The forehead provides a site with haemodynamic stability that allows the heart rate to be determined using the blood supply of the supraorbital artery and superficial temporal artery. These arteries are in turn supplied by the internal and external carotid arteries, which also supply blood to the brain. This makes the site much less susceptible to poor peripheral perfusion since blood flow to the brain is physiologically preserved at the expense of other less important organs and peripheral limbs (Schallom et al., 2007) (Berkenbosch and Tobias, 2006). Previous studies have recommended the forehead for neonatal monitoring due to its haemodynamic stability in comparison to the peripheries (Grubb et al., 2014). The forehead has also shown promise as a suitable location for non-contact PPG monitoring (Allen and Howell, 2014). A further advantage of forehead monitoring is that the wrists of neonates, especially preterm, are extremely small and it is not always possible to place more than one piece of equipment. Should a cannula be required, such as a peripheral venous line, PPG could not be performed on that wrist. By implementing PPG on the forehead, the wrists are available for cannulation where necessary.

We have previously studied a forehead-based PPG (fhPPG), operating in multiple newborn clinical settings (NICU and delivery room), demonstrating strong heart rate correlation with a gold standard device (Henry et al., 2020). The aim of this study is to compare a fhPPG sensor with a traditional, peripherally sited limb-based pulse oximeter for heart rate monitoring in more critically unwell newborns over a much longer study period (198 h compared to 16 h previously).

## 2 Materials and methods

### 2.1 Study population

This cohort observation study was conducted at the Nottingham University Hospitals NHS Trust, United Kingdom, following ethical approval (East Midlands - Nottingham 1 Research Ethics Committee 20/EM/0034). Informed parental consent was obtained prior to infants being recruited into the study. One of the aims of this trial was to evaluate the reliability and accuracy of the heart rate algorithm of the fhPPG device in critically unwell infants in the NICU. Infants were only recruited if they had an arterial line as part of routine care and required monitoring of vitals such as heart rate and blood oxygen saturation, the subject of a future publication with the same fhPPG sensor used here. Infants, regardless of gestational age or birthweight, were considered for the trial if they met the aforementioned criteria. The trial was terminated once the arterial line was removed from the infant. A convenience sample of 20 infants was recruited. One infant was excluded as there was no pulse oximeter (PO) data due to a lack of a research compatible monitor from which to collect data.

### 2.2 Study design

The aim of this study is to compare a forehead-based PPG (fhPPG) sensor with a traditional limb-based pulse oximeter for heart rate monitoring. We have previously shown a device operating in multiple clinical settings (NICU and delivery room) demonstrating strong correlation with a gold standard (Henry et al., 2020). The device has now been further trialed on a much larger sample of data points (198 h compared to 16 h previously) from critically unwell infants in neonatal intensive care to further assess the ability to measure heart rates quickly and accurately over a long continuous period.

**FIGURE 1:**
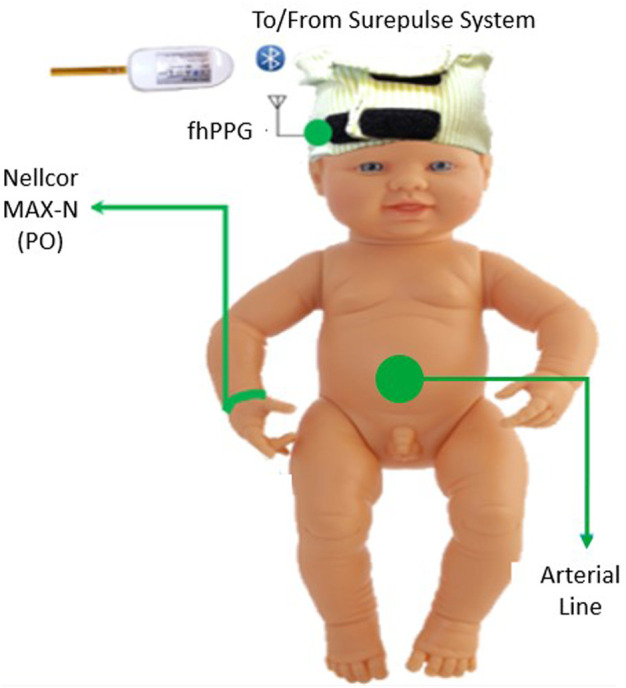
Diagram of the placement of the equipment on the subject in the trial. The Nellcor PO was placed on the right wrist and the fhPPG on the forehead.

For monitoring of the infant, the equipment consisted of an fhPPG device (Surepulse Medical Ltd.) housed inside a cap of appropriate size for the infant’s head and connected wirelessly to a data logging system *via* an inbuilt Bluetooth module. The probe has three LEDs of different wavelengths in the green (λ = 525 nm), red (λ = 660 nm) and IR (λ = 950 nm) regions. For the detection and calculation of heart rate, the fhPPG uses a green LED with a photodiode to detect changes in the magnitude of detected light as blood is pumped around the body. As part of routine care, a transmission mode Nellcor MAX-N PO was placed on the infant’s right wrist and was attached to a CARESCAPE Monitor B850 (General Electric Healthcare). Custom software designed in MATLAB 2021b (MathWorks) was used to collect synchronised data in real-time from the B850 and stored on a laptop (Lenovo Thinkpad L540, Lenovo Group Ltd.) running Windows 10. In accordance with regular practice, if either of the devices presented a poor or no trace, the device was repositioned in an attempt to restore the signal. This was performed by neonatal nurses when the trace observed on either the B850 or fhPPG display was noticeably poor as is normal care practice. A diagram of the equipment used, and site location is illustrated in Figure 1.

Each subject had multiple data recording sessions taken with the fhPPG device with each record approximately 60–90 min long. After each recording, the sensor was removed and placed back on the infant’s head. This removal was necessary to check the skin integrity under the sensor regarding redness and intact skin. Minimal redness was observed and importantly, there was no skin damage. The device has already met regulatory requirements for biocompatibility, however, additional safety measures were taken proactively to ensure the health of the subjects.

The raw fhPPG data is initially filtered in hardware by a 30 Hz single pole switched capacitor filter. It is then further filtered in software by a 3^rd^ order linear phase bandpass filter (0.4—9 Hz) to isolate the pulsatile signal of the PPG which has an expected frequency between 1 and 4 Hz (60bpm—240bpm). The linear phase response results in negligible distortion to the shape of the pulse compared to non-linear phase responses (Liu et al., 2021).

Prior to the beginning of each record, the clocks of both the fhPPG and the B850 were aligned to synchronise the two data streams. The B850 monitor generates heart rate data over a 10-s window with an update rate of one second. The fhPPG was set to the same window length as the B850. However, the fhPPG produces a heart rate value every 5 s. As such, the B850 heart rate data was downsampled to match the fhPPG, such that each device observes the same period for each window. Downsampling was chosen so that the time window observed by both devices matched exactly as opposed to averaging the B850 data.

### 2.3 Data analysis

Data analysis was performed using MATLAB. All continuous variables were tested for normality with the Lilliefors test and presented as mean and standard deviation (SD), median (range), or median (IQR), as appropriate. On occasions, signal integrity errors occurred with the B850 causing the data stream to output constant values for the heart rate for the PO. Where this occurred, or an error code was present, the data were disregarded and not included in the analysis.

For all data pairs (fhPPG and PO), the following output statistical values are calculated.

i) Positive percentage agreement (PPA)
ii) Bland-Altman plots (Limits of Agreement (LOA) and Bias)
iii) Spearman’s Correlation Coefficient (ρ)

When calculating the PPA, Spearman’s correlation coefficient, and Bland-Altman statistics, *“unsuccessful”* paired data points were removed from the analysis. An unsuccessful paired data point is where either the fhPPG or the PO device: is unable to output a reading due to an inability to calculate a heart rate; or a device presented an error code; or device outputs a heart rate value outside the standard operating ranges of 30–240 bpm. The removal of these data points reduced the total amount of data available to process by 25 h, from 223 h to 198 h of paired data, for a total of 142,567 data points.

For PPA, an fhPPG data point was considered in positive agreement if the data point was within 10% of the paired PO data point. This term provides a reliability indicator with an element of accuracy compared to the PO. Correlation between fhPPG heart rate and PO heart rate is shown with the Spearman’s correlation coefficient. Spearman’s correlation coefficient was chosen since the two values, fhPPG heart rate and PO heart rate are not independent of each other, as they are both attempting to measure the same signal. Finally, a comparison of fhPPG and PO heart rate values was performed by Bland-Altman analysis (Bland and Altman, 2007) reporting LOA and bias. The modified Bland-Altman analysis was selected due to the differing number of data points for each subject (Bland and Altman, 2007). For Spearman’s correlation coefficient, the value of ρ is calculated by taking the entire cohort of data. This is similar to the bias calculation from Bland & Altman, 2007 as it accurately weights the data based on the number of data points collected from each subject. The same cohort technique was also performed for the PPA, such that those subjects contributing the most data points to the analysis were weighted correctly.

## 3 Results

The demographics of the 19 subjects are shown in Table 1. A total of 187 recordings were made (median number of records 6/infant, range 1-34). This corresponds to a median total record length/infant of 7.16 h (IQR 4.12–17.05 h) with a minimum of 0.79 h and a maximum of 45.08 h/infant. In total 223 h of recordings were undertaken with a total of 198 h of paired data being analysed.

**TABLE 1:** Demographics of the study. All values are either absolute values or median and interquartile ranges (IQR). Primary reason for admission to NICU given.

Demographics and characteristics	Overall cohort (N = 19)
Gestational age at birth (weeks + days)	37^+2^ (27^+5^–40^+3^)
Chronological age at recruitment (days)	2 (1–12)
Birthweight (g)	2920 (1060–3610)
Male sex, n (%)	7 (37)
Fitzpatrick skin type, n (%)	
II	16 (84)
IV	2 (11)
V	1 (5)
Number of fhPPG records per subject	6 (1–34)
Principal cardiorespiratory diagnosis for neonatal admission, n (%)	
Persistent pulmonary hypertension	9 (47)
Congenital diaphragmatic hernia	3 (16)
Sepsis	2 (11)
Hydrops fetalis	2 (11)
Respiratory distress syndrome	1 (5)
Congenital heart defect	1 (5)
Right pneumothorax	1 (5)
Inotrope requirement, n (%)	12 (63)
Death before neonatal discharge, n (%)	1 (5)

**FIGURE 2:**
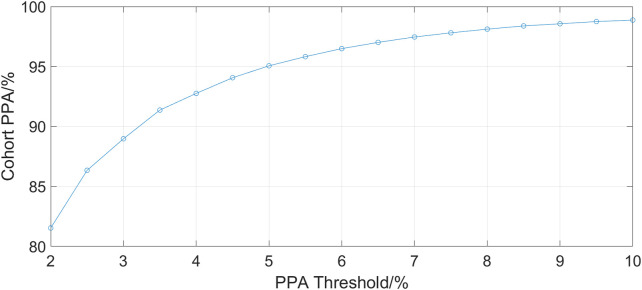
A plot of PPA threshold against the cohort PPA value. The PPA threshold was defined as the fhPPG heart rate being within a certain percentage of the PO heart rate.

**FIGURE 3:**
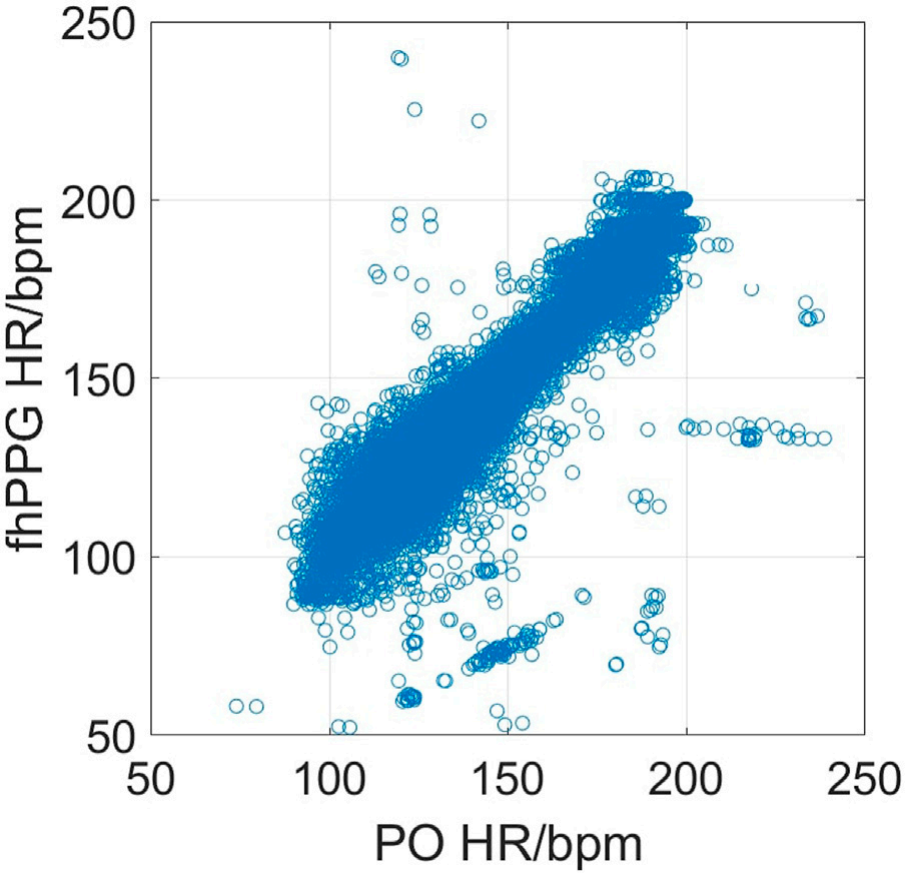
Scatter plot of heart rate between PO vs. fhPPG (n = 142,567) for 19 subjects.

**FIGURE 4:**
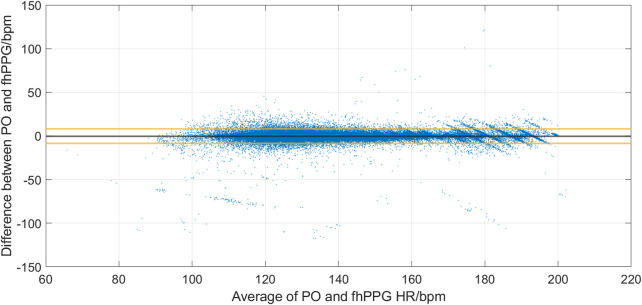
Bland-Altman analysis of PO and fhPPG (n = 142,567) for 19 subjects.

Figure 2 shows the cohort PPA against PPA thresholds in the range of 2%–10%. At a threshold of 10% (where the fhPPG heart rate was within 10% of the PO heart rate), the cohort had a PPA of 98.87%. Pearson’s correlation coefficient had a strong correlation of 0.9816 (*p* < 0.001) between the PO heart rate and fhPPG heart rate. Bland-Altman analysis of the data demonstrated a small negative bias of −0.22 bpm with limits of agreement of 8.44 bpm. All fhPPG and PO pairs used in analysis are shown in a pooled scatter plot and Bland-Altman plot in Figures 3, 4 respectively.

Of the 19 subjects recruited, eight were considered preterm (gestation age (GA) < 37 weeks). Of these eight, five were moderate to late preterm (GA 32–37 weeks), two are very preterm (GA 28–32 weeks) and one is extremely preterm (GA < 28 weeks). Table 2 shows the measurement statistics of two sub-cohorts of the data, term subjects and preterm subjects.

**TABLE 2:** Accuracy results when separated into sub-cohorts of term and preterm infants. Spearman’s correlation coefficient (ρ), bias and LOA are presented.

Cohort\Variable	N	ρ	Bias (bpm)	LOA (bpm)
Cohort	19	0.9816	−0.22	8.44
Term	11	0.9819	−0.17	6.99
Preterm	8	0.9750	−0.46	12.41

## 4 Discussion

The aim of this trial was to assess the accuracy and reliability of a head mounted fhPPG heart rate sensor on a large cohort of data from critically ill infants not previously studied (Henry et al., 2020). This was achieved as the dataset collected was over ten times larger, with over 198 h of data compared to 16 h, albeit with only 19 subjects compared to 34 previously. The strongly positive Spearman’s correlation coefficient shows that the fhPPG was able to track the changes in the heart rate when compared with the PO. When both devices were working in tandem, a very high PPA was achieved when tested at all thresholds. This suggests that the fhPPG consistently calculates heart rate to within a small tolerance of the PO, with 98.87% of fhPPG heart rate datapoints in positive agreement with the PO at a 10% threshold. Given that the heart rate of the subjects was generally between 100 and 200 bpm, the 81.53% PPA at a 2% threshold shows that the fhPPG was within 2–4 bpm of the PO over 80% of the time. The high PPA, in combination with the strongly positive Spearman’s correlation coefficient, suggests that the fhPPG is not only able to track changes in heart rate but also to do so accurately.

Previously, work has been undertaken to find the best location for a pulse oximeter. Longmore et al. found that if only heart rate and SpO_2_ is required, then the forehead is the most suitable location with the smallest median error compared to a finger-based PPG sensor (Longmore et al., 2019). Furthermore, Peralta et al. (2017) showed that the forehead provided greater accuracy than the finger when detecting pulse rate variability, a derivative of the heart rate. However, along with most forehead PPG comparison studies, these tests were performed on adults. The previous work on reflection-mode monitoring in newborns compared ECG (Grubb et al., 2014; Henry et al., 2020) or PPG from a non-forehead site such as the thigh (Johansson et al., 1999).

Whilst the magnitude of the bias decreased from the previous trial (Henry et al., 2020) (−0.22 bpm vs. 0.6 bpm) there was an increase in the LOA (8.44 bpm vs. 5 bpm). The aim of this trial was to compare two optical based devices at different locations on the body, forehead and wrist, in critically unwell subjects. However, the use of a wrist-based PO is a limitation as comparison to the more accurate ECG heart rate as suggested by ISO 80601-2 (BS EN ISO 80601-2-61:2019) is recommended. ECG probes were placed on the subject in line with standard care practice, however ECG data for the whole dataset was unavailable for comparison. The previous analysis of accuracy statistics (RMSE and Bland-Altman) was conducted against ECG (Henry et al., 2020) and is a possible reason why the LOA has increased when compared with the previous data. Another potential reason for the increase in the LOA in preterm cohort is that the infants were on multiple vaso-active medications, such as adrenaline and ventilation modalities that could interfere with the PPG signal from either device. As these infants were much sicker than those previously studied (Henry et al., 2020), these data indicate that the fhPPG can still accurately monitor critically ill infants with only small reduction in accuracy.

This study enrolled subjects of varying gestational age. Of the 19 subjects enrolled, 11 were term and eight preterm infants. For the preterm infants, there was an increase in the bias and LOA when compared with the term infants. The bias remained small, however the LOA increased by 5.42 bpm to 12.41 bpm. This is likely due to a variety of factors including the higher baseline heart rate of preterm infants, often increased further with caffeine treatment, and the differing pathologies between term and preterm infants, such as an underdeveloped respiratory and circulatory system. As a result, the ratio of the noise and motion artefacts compared to the measured PPG signal in newborns is larger compared to adults showing the importance of such a new medical device for paediatricians use and provides a benchmark for future improvements. The Spearman’s correlation coefficient remained strongly positive however at 0.975, suggesting that the fhPPG was able to track the changes in heart rate but the fhPPG and PO disagreed on the value of the heart rate more so than in term infants. As discussed previously this could be due in part to errors in the PO as opposed to errors in the fhPPG heart rate, or a combination of errors in both devices.

## 5 Conclusion

The results presented demonstrate that the forehead is an equally suitable site for measuring reflectance mode PPG for heart rate compared to a peripheral transmission mode PO. The use of the forehead for monitoring PPG signals for heart rate provides benefits when compared with peripheral limbs. The forehead allows a measurement of the core blood supply which is less susceptible to decreases in perfusion during times of stress (Berkenbosch and Tobias, 2006). Additionally, it opens up the possibility to use the wrists or ankles for other interventions, such as cannulas. By coupling these advantages with the ability to wirelessly transmit data to a remote screen, forehead PPG also increases newborn accessibility allowing vital skin to skin parental bonding and easier clinical access to the infant. Further work investigating the effects of different therapies and drugs on neonates would help assess further the benefits of the forehead compared with the wrist for PPG monitoring.

## Data Availability

The datasets presented in this article are not readily available for public access as it will be further used for commercial purposes by Surepulse Medical Ltd. The data will be made publicly available at a future date, please contact the authors. Requests to access the datasets should be directed to Barrie Hayes-Gill, barrie.hayes-gill@nottingham.ac.uk.
